# Minimizing control group allocation in randomized trials using dynamic borrowing of external control data – An application to second line therapy for non-small cell lung cancer

**DOI:** 10.1016/j.conctc.2019.100446

**Published:** 2019-09-09

**Authors:** Louis Dron, Shirin Golchi, Grace Hsu, Kristian Thorlund

**Affiliations:** a Department of Health Research Methodology, Evidence, and Impact, McMaster University, Hamilton, ON, Canada; b Department of Statistics and Actuarial Science, Simon Fraser University, Burnaby, BC, Canada; c MTEK Sciences, Vancouver, BC, Canada

**Keywords:** Synthetic control, NSCLC, Oncology, Bayesian hierarchical model, Dynamic borrowing, External control

## Abstract

**Background:**

Enrollment of participants to control arms in clinical trials can be challenging. This is particularly an issue in oncology trials where the standard-of-care is shifting rapidly and several promising experimental treatments are undergoing phase III testing. Novel methods for utilizing external control data may mitigate these challenges, but applied examples are sparse. Here, we therefore illustrate how Bayesian dynamic borrowing of external individual patient level control data from similar clinical trials can often reduce randomization to the control intervention without substantially trading-off precision. We further explore which types of scenarios yield viable trade-offs, and which do not.

**Patients and methods:**

We obtained individual patient data on patients being treated with second-line therapy for non-small cell lung cancer from Project Data Sphere with minimal in/exclusion criteria restrictions, and applied Bayesian hierarchical models with uninformative priors to generate illustrative synthetic control groups.

**Results:**

Four phase III clinical trials were identified and utilized in our analysis. Even when studies which are knowingly incongruent with one another are selected to generate a synthetic control, the nature of this methodology minimizes improper borrowing from historical data. The use of a small concurrent control group within a trial greatly reduces penalized selection, and our results demonstrate the ability to reduce allocation to the control group by up to 80% with a minimal increase in uncertainty when closely matched historical data is available.

**Conclusion:**

Dynamic borrowing using Bayesian hierarchical models with uninformative priors represents a novel approach to utilizing external controls for comparative estimates using single arm evidence.

## 1 Introduction

Clinical efficacy research for medical interventions is evolving rapidly, and methods to study rare disease and patient subgroups in particular have undergone significant improvements in recent years [1]. Despite this, assignment to control groups in this context create particular ethical and efficiency challenges, by ‘wasting’ limited target sample sizes on enrolling a substantial proportion of patients to a control intervention [2]. These issues are not limited to rare diseases or patient sub-populations only, but represent a concern for all randomized controlled clinical research [3]. Fear of being given placebo has been given as a top reason as to why patients feel clinical research may not be safe [4].

One approach to manage these concerns is to better integrate external evidence into clinical efficacy assessments. Numerous regulatory and reimbursement agencies have indicated an openness to exploring the use of external, or ‘real-world’, data as a substitute of support for absent or sparse control group data for comparative efficacy research. The FDA is joined by multiple international agencies, academic groups and think-tanks [[5], [6], [7]] who advocate for improved integration of non-randomized evidence into comparative efficacy analytics. This is becoming reflected in regulatory submissions for new treatments, although over half (64%) of submissions to the National Institute of Clinical Excellence (NICE) which utilized non-comparative data as part of their submission relied on naïve unadjusted external controls [8].

Sole reliance on external controls remains sub-optimal, as without a concurrent control group it is not possible to determine what, if any, differences exist between the historical control population and the active trial [9]. As such, many research groups have advocated for trial designs that recruit a concurrent control group at an unequal randomization ratio (e.g., 1:3 between control and intervention), and incorporate external control data to increase the precision of the synthetic control group (i.e. the combination of concurrent and external control data) to that of the intervention group [10,11,16]. Two immediate challenges that arise from this approach is whether the external data introduces meaningful confounding on the synthetic control response or whether the heterogeneity between external control sources is so large that the effective added sample size becomes negligible. Many previous applications have proposed subjectively excluding or down weighting ‘heterogeneous’ control sources. Recently, Bayesian ‘dynamic borrowing’ models have been proposed as a flexible and non-subjective solution to these issues. With this approach, external control sources are statistically down weighted proportionally to the degree of heterogeneity it introduces, thereby both ensuring minimal introduction of confounding and optimization of the effective sample size from the external control source(s). While this approach holds great promise, more applications are needed to further understand their advantages and pitfalls.

Here, we present an example of dynamic borrowing of external control data from a selection of trials in second line non-small cell lung cancer (NSCLC), applied to overall survival. We demonstrate how our dynamic borrowing methodology can produce reliable estimations of comparative treatment efficacy, while mitigating the need for enrollment to control intervention. Further, we demonstrate the importance of data selection, by providing the results of analyses conducted with both homogeneous and heterogeneous external data and illustrating the influence of the data source on the accuracy and precision comparative treatment effect estimates.

## 2 Methods

### 2.1 Data source and study selection

Data used in our model was acquired from Project Data Sphere (http://www.projectdatasphere.com), an open-source repository of individual-level patient data from phase IIB/III oncology trials. As our methodology required individual-level patient data, we investigated which available trials in a sufficiently similar patient population were available on the system. We filtered results by major tumor type, restricted results to phase III trials and excluded any trials with multiple tumor types. No restrictions were used for interventions, year of publication or cancer stage. After adjustment for line of therapy, we identified that second line NSCLC had the greatest number of trials suitable for our analysis.

Data was downloaded in the standardized study data tabulation model (SDTM) format for each publication alongside its’ associated data dictionary. Verification of data was achieved by ensuring that patient numbers and event rates corresponded to peer-reviewed published literature estimates.

### 2.2 Types of borrowing scenarios considered

Borrowing information from past studies naturally relies on the assumption that the concurrent and historical trials are sufficiently similar. In practice, however, this is not guaranteed. To illustrate the impact of relaxing or tightening the criteria on which this similarity assumption is based, we considered two types of borrowing: ‘low-risk’ and ‘high-risk’ borrowing.

#### 2.2.1 ‘Low-risk’ borrowing

For the analysis using ‘low-risk’ borrowing, we selected the INTEREST [12] trial as the concurrent trial. We specifically selected this trial due to its similarity of overall survival (OS) control arm results relative to the ZODIAC [13] and Study 57 [14] trials, but also owing to relative similarities with regards to histology, prior lines of treatment and lack of radiotherapy. This scenario represents a scenario wherein there are multiple comparable trials covering a similar patient population with comparable clinical outcomes.

#### 2.2.2 “High-risk” borrowing

For the analysis using ‘high-risk’ borrowing, we selected the PROCLAIM trial [15] as the concurrent trial. We specifically selected this trial due to its control arm OS being substantially different from the other three trials, alongside significant discrepancies with regards to tumour stage and delivery of concurrent chemoradiotherapy. This scenario represents a hypothetical scenario where relatively well-matched historical control data is unavailable, and therefore historical trials of moderate similarity are utilized as an attempt to generate a synthetic control.

### 2.3 Dynamic borrowing using Bayesian hierarchical models with informative priors

The model is essentially equivalent to a mixed effect model where control response of the individual trial is incorporated as a random effect. The use of historical control data is through an uninformative prior distribution defined on the parameter of the concurrent control arm.

Specifically, concurrent control refers to participants actively and prospectively recruited for a given trial. The amount of information borrowed from the historical data is determined dynamically through a Bayesian hierarchical model. This approach is based on a mixed effect model with the assumption that the baseline parameter of the concurrent study $\theta_{c}$and the baseline parameters of the H historical studies $\theta_{h}$, h=1,…,H, follow a Gaussian distribution whose mean and variance are inferred by the data. That is,

$$\theta_{c},\;\theta_{h}\sim Normal\left(\mu ,\;\tau \right),\;\;\;\;h=1,\;\ldots ,\;H,\;\;\tau >0$$

where μ andτ are assigned uninformative priors and τ is truncated to be greater than zero. The time-to-event data is modelled by a Weibull distribution. The regression model, including the baseline parameters, covariates and treatment effects is embedded as fixed-effects in the Weibull distribution's scale parameter (see Appendix A for full details on the model specification).

### 2.4 Considerations for selection of historical control data

The risk in borrowing information from previous studies comes from borrowing from trials dissimilar to the concurrent trial in terms of prognostic factors and response to the treatment, which may result in misleading the inference and thereby penalized estimates of the treatment effect.

The control responses, whether concurrent or external, are modelled as a standard random-effect. As such, when a large degree of heterogeneity exists between control responses, the credible intervals for the comparative treatment effect widen. In contrast, a mild-to-moderate degree of heterogeneity allows for a meaningful improvement in precision compared to that of only using a concurrent control.

### 2.5 Interactions between prior information and trial randomization

**Fig. 1:**
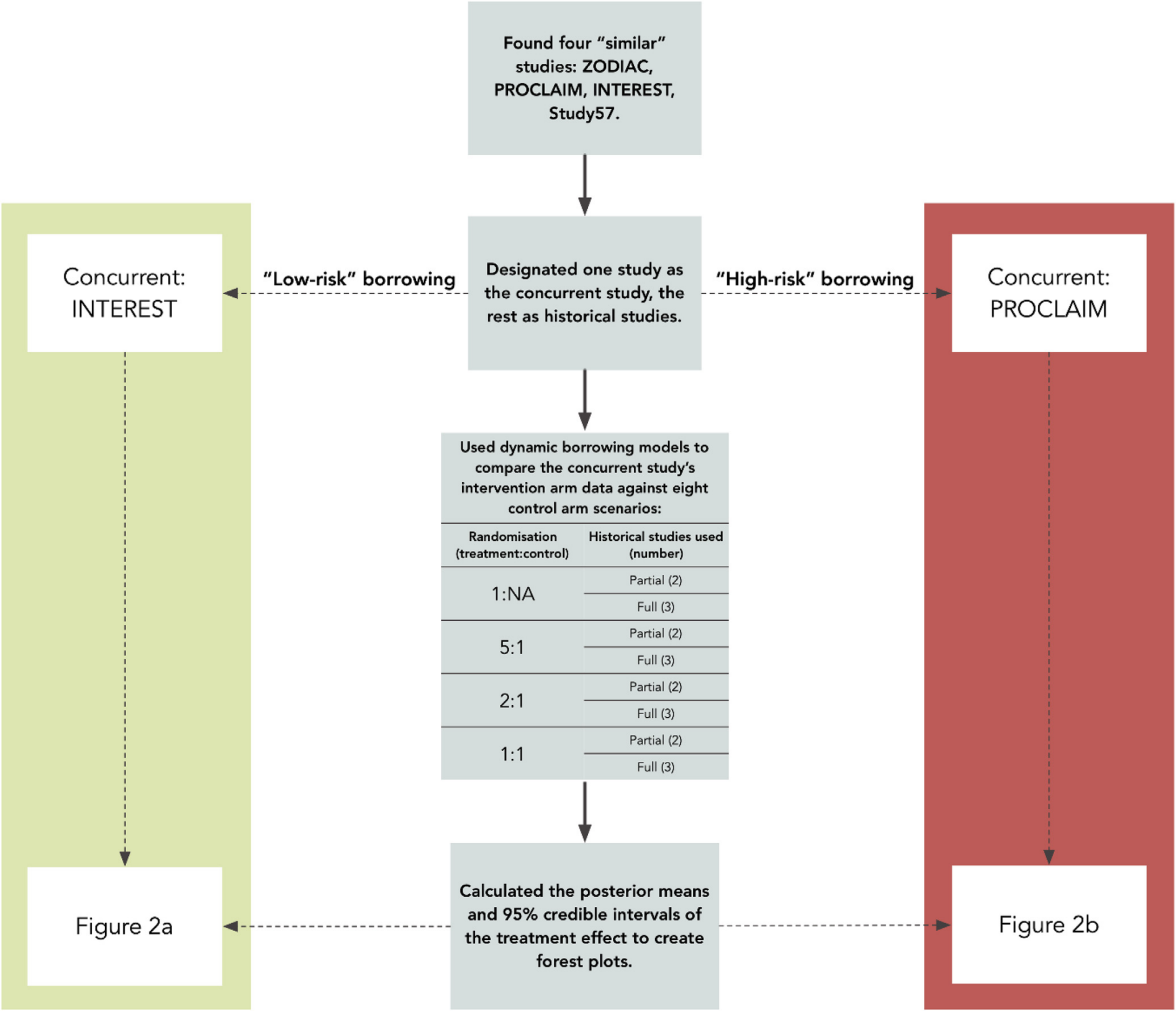
A flowchart of how included trials were incorporated into our analysis, and how their data was utilized.

The amount of information borrowed under the dynamic borrowing model is determined by various factors: The number and size of historical studies, the size of the control arm in the concurrent study, the variability among the historical studies and the discrepancy between the historical and concurrent control data. We therefore considered a number of scenarios that we consider to be either ‘low-risk’ or ‘high-risk’ borrowing. For the construction of priors, we considered using either the two most similar of the available historical controls, representing low variability among historical data, or all three controls. Under the ‘low-risk’ borrowing scenario, the PROCLAIM trial was excluded from the historical control data set for the analytical setting where only two trials were used. Under the ‘high-risk’ scenario, Study 57 was excluded from the historical control data. We further considered a number of scenarios for the randomization ratio between the experimental intervention arm and the control arm. In particular, we considered the option of 1:1, 2:1, 5:1 randomization as well as a design where no patients were randomized (1:NA) to the control arm. Fig. 1 outlines all analysis and design approaches tested under the ‘low-risk’ and ‘high-risk’ borrowing scenarios.

## 3 Results

### 3.1 Included data

We identified four trials with relevant data for our analysis, INTEREST [12], ZODIAC [13], PROCLAIM [15], and Study 57 [14]. All trials were conducted for participants who had previously been treated for NSCLC. A brief summary of key trial characteristics for the included trials is provided in Table 1. Sample size was similar across included trials, with the exception of PROCLAIM, which was terminated early owing to futility. Patients were predominantly stage IV, however PROCLAIM exclusively recruited patients who were stage III (of which 52% were stage IIIB). This is reflected in the median overall survival time, which was between 8 and 10 months for all trials except PROCLAIM, which demonstrated a control group median survival time of 25 months. Exposure to prior therapy varied. Whilst all included patients had previously received at least one prior chemotherapy regimen, the proportion of patients who had received two or more varied between 0 (PROCLAIM, ZODIAC) to 35% (Study 57). Similarly, radiotherapy varied significantly, with PROCLAIM being the only trial which permitted (concurrent) chemoradiotherapy. Other patient characteristics were largely well balanced. Of note, the INTEREST trial was designed to be a non-inferiority study, as opposed to the other publications which were designed as superiority trials.

**Table 1:** A summary of patient and trial characteristics for included publications.

Characteristic	INTEREST	ZODIAC	PROCLAIM	Study 57
Overall sample size (*n*)	1433	1391	598	1240
Control group median overall survival, months (Q1, Q3)	8 (4, 14)	10 (4, 13)	25 (10, 35)	8 (4, 12)
Stage III, (%)	38	15	100	17
Stage IV, (%)	53	85	0	83
Average age, (years)	61	59	59	61
Adenocarcinoma histology, (%)	54	60	75	60
Two or more prior chemotherapy regimens, (%)	16	0	0	35
Radiotherapy sequence, dose, (control arm)	None	None	60–66Gy, Concurrent	None

In summary, the PROCLAIM was substantially different that the two others with a median survival average over twice that of other trials, the exclusion of any stage IV patients and the possible incorporation of concurrent chemoradiotherapy as part of the treatment regimens.

### 3.2 Findings

#### 3.2.1 ‘Low-risk’ borrowing

**Fig. 2:**
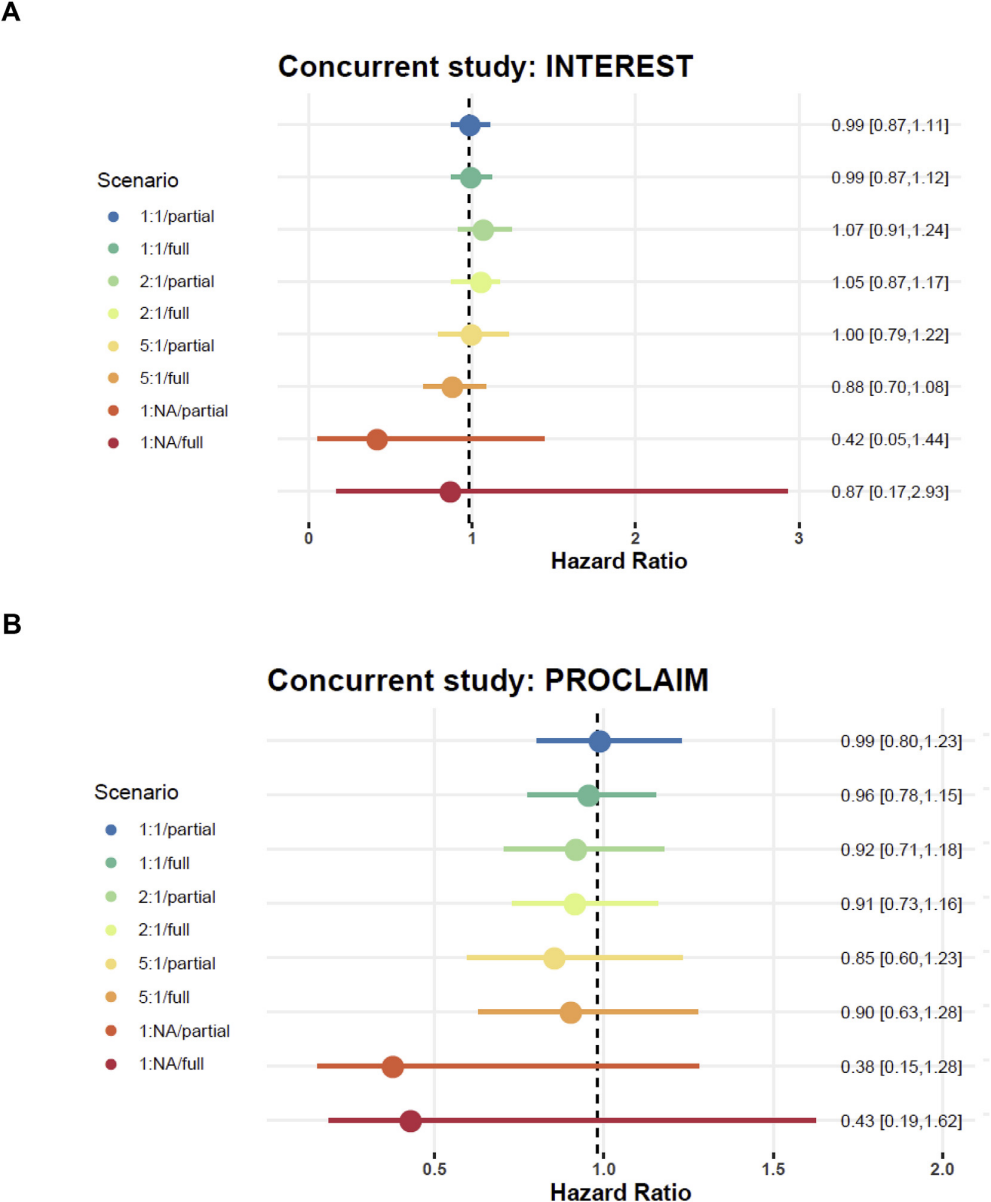
a: Point estimates and 95% credible intervals for the hazard ratio obtained by the dynamic borrowing model using full/partial historical control and various portions of the concurrent control data for the INTEREST (low risk) trial. The vertical line shows the reported estimate of the hazard ratio. Fig. 2b shows the same as Fig. 2a, but with concurrent control data from the PROCLAIM (high risk) trial.

Fig. 2a shows the results as point estimates (posterior mean) of the hazard ratio with 95% credible intervals for differing designs and analysis settings when INTEREST is the control trial of interest. When a reasonable portion of the concurrent control data is used (1:1 and 2:1 randomization) using two versus three trials as historical control data does not make any notable difference. The estimates of hazard ratios under these scenarios are very close to the reported values, with 1:1 (full) generating a hazard ratio of 0.99 (95% CrI: 0.81–1.11), and 2:1 (partial) generating a hazard ratio of 1.05 (95% CrI: 0.81–1.17). In case of the 5:1 randomization, however, excluding PROCLAIM helps improve the results, changing the hazard ratio estimation from 0.88 (95% CrI: 0.70–1.08, 5:1/full) to 1.00 (95% CrI: 0.79–1.22, 5:1/partial), owing to differences in PROCLAIM relative to other included trials in terms of control group survival times. It is not surprising that there is substantial inflation in uncertainty when no concurrent control data is used – the situation is yet even more extreme if all the historical studies including PROCLAIM is used in the inference.

#### 3.2.2 “High-risk” borrowing

Fig. 2b shows the estimates and 95% credible intervals of the hazard ratios for differing designs and analysis settings when PROCLAIM is the control trial of interest. Although this example is to represent the case where the concurrent study is different than the historical control and therefore there is a risk of penalized parameter estimates, the results are reassuring in that like in “low-risk” borrowing the 95% credible intervals always include the value of 1. Of course, the estimates depart from the reported hazard ratios as the concurrent control data gets smaller and there is little information to correct for the discrepancy between the concurrent and historical data. Using two versus three historical studies makes no noticeable difference in this case since the three historical studies are fairly similar and using a subset instead of all does not imply lower variation, unlike the previous example. For example, at a 5:1 randomization, three studies provide a hazard ratio of 0.90 (95% CrI: 0.63–1.28, 5:1/full) as opposed to a hazard ratio of 0.85 (95% CrI: 0.60–1.23, 5:1/partial) with two historical studies.

## 4 Discussion

In this paper we investigated the dynamic borrowing model, which uses historical data to empower inference in clinical trials where a control arm is either absent or contains insufficient data due to ethical issues. To explore various scenarios in borrowing information from historical studies, for each of the examples presented, we consider one of the trials as the ongoing trial and the remaining trials as historical studies whose control arms are to be used to inform the treatment effect inference. The included examples represent two frequently encountered scenarios where the concurrent trial is similar to at least a portion of the available historical studies, and the case that it is different than the past data and therefore borrowing information requires caution.

The main results can be summarized as follows: Little difference in terms of the final conclusions is made when different subsets of the past studies are used – especially if the historical studies are similar and/or sufficient data are available under the concurrent trial control arm. Having less information (patients randomized) under the concurrent trial control arm results in higher levels of uncertainty. Penalization of estimated parameters can arise in cases where not enough data is available to inform the model of discrepancy between the concurrent and historical studies.

The main advantage of the dynamic borrowing approach is that the amount of information borrowed is determined by the variability among the historical studies as well as the discrepancy between the concurrent and historical data. In other words, the level of borrowing is decided by the data through a hierarchical Bayesian mixed effects model rather than subjectively by the researcher. The main implication is that as long as there is some data available under the concurrent trial control arm, the risk of penalized conclusions is low as the model is able to identify potential discrepancies. For a single arm trial (no concurrent control arm), however, the model relies solely on the historical studies to estimate the prior uncertainty. Therefore, in such cases, it is recommended that as much historical data as is available be used such that, in case of deviance between the historical and ongoing studies, underestimating the posterior uncertainty and drawing incorrect conclusions is avoided. Note that the results of this paper may be limited to the structure of the data at hand, i.e., three similar and one different study. Having access to a larger collection of studies with different levels of heterogeneity can reveal other features of the dynamic borrowing approach that is beyond the scope of this work.

Our work demonstrates one approach to minimizing both analytical uncertainty and potential researcher's penalization in selection of trials in a transparent and data-driven framework.

## 5 Conclusions

Dynamic borrowing using Bayesian hierarchical models with informative priors is a methodology which enables a data-driven approach to synthesizing historical data, which can be used as a reference for trials with limited or non-existent control data.

## Disclosure

All authors report no conflicts of interest in this work.

## Funding

This research did not receive any specific grant from funding agencies in the public, commercial, or not-for-profit sectors.

## References

[1] Gagne J.J., Thompson L., O'Keefe K., Kesselheim A.S. (2014). Innovative research methods for studying treatments for rare diseases: methodological review.

[2] Gaddipati H., Liu K., Pariser A., Pazdur R. (2012). Rare cancer trial design: lessons from FDA approvals. Clin. Cancer Res..

[3] Nardini C. (2014). The ethics of clinical trials. Ecancermedicalscience.

[4] CISCRP (2017). Report on General Perceptions and Knowledge on Clinical Research.

[5] NAo Sciences (2018). The national academies collection: reports funded by National Institutes of Health. Examining the Impact of Real-World Evidence on Medical Product Development: I. Incentives: Proceedings of a Workshop-In Brief.

[6] Garrison L.P., Neumann P.J., Erickson P. (2007). Using real-world data for coverage and payment decisions: the ISPOR Real-World Data Task Force report. Value Health.

[7] Berger M., Daniel G., Frank K. (2017). A Framework for Regulatory Use of Real-World Evidence.

[8] Anderson M., Naci H., Morrison D. (2018). A review of NICE appraisals of pharmaceuticals 2000-2016 found variation in establishing comparative clinical effectiveness. J. Clin. Epidemiol..

[9] Jarow J. (2017). Use of external controls in regulatory decision-making.

[10] Viele K., Berry S., Neuenschwander B. (2014). Use of historical control data for assessing treatment effects in clinical trials. Pharm. Stat..

[11] Neuenschwander B., Capkun-Niggli G., Branson M., Spiegelhalter D.J. (2010). Summarizing historical information on controls in clinical trials. Clin. Trials.

[12] Kim E.S., Hirsh V., Mok T. (2008). Gefitinib versus docetaxel in previously treated non-small-cell lung cancer (INTEREST): a randomised phase III trial. Lancet.

[13] Herbst R.S., Sun Y., Eberhardt W.E.E. (2010). Vandetanib plus docetaxel versus docetaxel as second-line treatment for patients with advanced non-small-cell lung cancer (ZODIAC): a double-blind, randomised, phase 3 trial. Lancet Oncol..

[14] Natale R.B., Thongprasert S., Greco F.A. (2011). Phase III trial of vandetanib compared with erlotinib in patients with previously treated advanced non-small-cell lung cancer. J. Clin. Oncol..

[15] Senan S., Brade A., Wang L.H. (2016). PROCLAIM: randomized phase III trial of pemetrexed-cisplatin or etoposide-cisplatin plus thoracic radiation therapy followed by consolidation chemotherapy in locally advanced nonsquamous non-small-cell lung cancer. J. Clin. Oncol..

[16] Hobbs B.P., Carlin B.P., Mandrekar S.J., Sargent D.J. (2011). Hierarchical commensurate and power prior models for adaptive incorporation of historical information in clinical trials. Biometrics.

