# Supplementary material for: Minimizing control group allocation in randomized trials using dynamic borrowing of external control data – An application to second line therapy for non-small cell lung cancer
Source: Contemp Clin Trials Commun. 2019 Sep 9;16:100446. doi: 10.1016/j.conctc.2019.100446 (PMC6745532; doi:10.1016/j.conctc.2019.100446)
Supplement: Multimedia component 1 [file mmc1.docx]

# Appendix A: The model

The following Bayesian hierarchical model is used to analyze the data;

## **The likelihood function**

The likelihood function is defined as follows:

$$L\left( \alpha, \lambda\mid\boldsymbol{y}, \boldsymbol{\nu} \right)=\prod_{i=1}^{n} f\left( y_{i} \mid\alpha, \lambda\right)^{\nu_{i}}S\left( y_{i} \mid\alpha, \lambda\right)^{\left( 1-\nu_{i} \right)}$$

$$= \alpha^{d}exp\{d\lambda+ \sum_{i=1}^{n} {(\nu}_{i}\left( \alpha- 1 \right)\log\left( y_{i} \right)-\exp\left( \lambda\right)y_{i}^{\alpha})\}$$

Where $\boldsymbol{y}=(y_{1},\ldots, y_{n})$ is the vector of time-to-event observations, $\nu=\left( \nu_{1},\ldots, \nu_{n} \right)$ is the vector of censoring variables where $\nu_{i}=0$ indicates that $y_{i}$ is right censored and $\nu_{i}=1$ indicates otherwise. The $f(y_{i}\mid\alpha, \lambda)$ is the probability density function of a Weibull distribution with parameters $\alpha$ and $\lambda$ and $S(y_{i}\mid\alpha, \lambda)$ is the cumulative density function of the same distribution. In addition, $d= \sum_{i=1}^{n} \nu_{i}$.

For any given study the regression model is embedded within the scale parameter $\lambda$, i.e., for patient $i,$

$$\lambda_{i}=\theta+\boldsymbol{x}_{\boldsymbol{i}}\boldsymbol{\beta}+a_{i}\delta$$

where $\theta$ is the study-specific baseline parameter, $\boldsymbol{x}_{\boldsymbol{i}}$ is the covariate vector of patient $i$, $\boldsymbol{\beta}$ is the vector of covariate coefficients, $a_{i}$ is the treatment assignment indicator for patient $i$, and $\delta$ is the treatment effect. Therefore, the likelihood can be written as,

$L\left( \alpha, \theta, \boldsymbol{\beta},\delta\mid\boldsymbol{y}, \boldsymbol{\nu} \right)=\prod_{i=1}^{n} f\left( y_{i} \mid\alpha, \theta, \boldsymbol{\beta},\delta\right)^{\nu_{i}}S\left( y_{i} \mid\alpha,\theta, \boldsymbol{\beta},\delta\right)^{\left( 1-\nu_{i} \right)}$.

Under the above model the hazard ratio is given as,

$HR=\exp\left( \delta\right)$.

## **Defining the prior distribution**

The prior distribution for the model parameters is specified as the following,

$\theta_{c}, \theta_{h}\sim Normal\left( \mu, \tau\right),$ $h=1, \ldots, H$

where $\theta_{c}$ and $\theta_{h}$ are the baseline parameters of the concurrent and historical studies, respectively, and,

$$\mu, \delta\sim Normal\left( 0, 100 \right),$$

$\tau$ $\sim Normal\left( 0, 1000 \right), \tau>0,$

$\alpha$ $\sim Gamma(1, 1)$,

$\boldsymbol{\beta}$ $\sim Normal(0, 10)$.
